# lncRNA IL-17RA-1 Attenuates LPS-Induced Sepsis via miR-7847-3p/PRKCG-Mediated MAPK Signaling Pathway

**DOI:** 10.1155/2022/9923204

**Published:** 2022-10-13

**Authors:** Yuanxiu Gan, Jie Long, Yan Zeng, Yan Zhang, Yang Tao

**Affiliations:** Department of Critical Care Medicine, Chongqing University Central Hospital, Chongqing 400000, China

## Abstract

Sepsis represents a syndrome of systemic inflammatory response, which is mostly a result of infection with various pathogenic microorganisms, characterized by an uncontrolled infection response of the organism leading to life-threatening organ dysfunction. Long noncoding RNA (lncRNA), as competing endogenous RNA, can affect the binding of microRNA (miRNA) to mRNA, thus influencing the development of sepsis. In this study, based on transcriptome data from GEO database, we screened differentially expressed lncRNAs and constructed lncRNA-miRNA-mRNA network. And pathway IL-17RA-1/miR-7847-3p/protein kinase C gamma (PRKCG) coexpression network was successfully sorted out. The effect of this network on LPS-induced sepsis model in THP-1 cells was also verified by CCK-8, scratch, ELISA, Western blot, and qRT-PCR assays. Corresponding binding sites of miR-7847-3p to IL-17RA-1 and miR-7847-3p to PRKCG were verified using dual luciferase gene reporter assays, respectively. Compared with control, si-IL-17RA-1 significantly inhibited the cell viability and migration ability of THP-1, and levels of proinflammatory factors IL-6, IL-1*β*, and TNF-*α* secreted were markedly decreased, and the expression of IL-17RA-1, PRKCG, p-MEKK1, and p-JNK were markedly reduced. In addition, IL-17RA-1 could target binding to miR-7847-3p and inhibit its expression, and miR-7847-3p could also bind to PRKCG. Our experiments demonstrate that IL17-RA-1 attenuates the sepsis response through the miR-7847-3p/MAPK pathway, and this competing endogenous RNA (ceRNA) network may be a potential approach to predict and combat sepsis.

## 1. Introduction

Sepsis is an abnormal immune response to infection accompanied by organ dysfunction [1], mostly attributable to pneumonia, cholangitis, peritonitis, urinary tract infection, meningitis, cellulitis as well as abscess. The microorganisms associated with pathogenesis mainly include bacteria, fungi, viruses, and parasites. At present, the specific mechanism of sepsis has not been fully elucidated, and its progression covers diverse body response processes to diverse pathogenic microorganisms and their toxins such as immune dysfunction, intricate systemic inflammatory response, coagulation dysfunction, and tissue injury. Consequently, pathological changes occur in multiple systems and organs of the body, including injured kidneys, impaired lung function, and myocardial cell damage [2]. Sepsis has a high morbidity and mortality rate. Globally, nearly 50 million cases of sepsis occur each year [3], and septic shock caused by sepsis can lead to death in up to 40% of hospitalized patients [4]. Delayed sepsis management entails irreversible organ damage and organ function failure, and clinical mortality is positively correlated with delayed disease management, which is as high as 90% or above when sepsis affects four to five organs [5]. As sepsis occurs and progresses rapidly, the exploration of the mechanism of organ damage due to sepsis lays a solid foundation for taking effective prevention and therapeutic measures in clinical settings.

Long noncoding RNAs (lncRNAs) are a class of transcripts that lack protein-coding capacity. lncRNAs join several cellular processes namely proliferation, apoptosis, migration, and establishment of cellular properties [6]. Studies have found that lncRNA can have sponge adsorption to microRNA (miRNA), and it competitively binds to miRNA as competing endogenous RNA (ceRNA), thereby affecting to some extent miRNA regulation of mRNA to reduce organ damage caused by sepsis [7–9]. Although some studies have preliminarily explored the mechanism of lncRNA-induced organ damage, due to the wide variety of sepsis-related lncRNAs with little understanding, further exploration is also of vital significance.

GEO database in the research was applied to screen differentially expressed lncRNAs and miRNAs in sepsis, and the genes closely related to clinical features were screened out. Additionally, we combined cell tests to verify the predicted results to provide reference for the early clinical diagnosis, treatment options, and prognosis of sepsis.

## 2. Methods

### 2.1. Data Download

GEO database (https://www.ncbi.nlm.nih.gov/geo/) associated with sepsis datasets was searched and retrieved including three datasets, namely, mRNA dataset (GSE201958), miRNA dataset (GSE137294), and lncRNA dataset (GSE145227).

### 2.2. Differential lncRNA Analysis

The downloaded data were analyzed for variance via the R software limma package. *p* values were obtained and corrected for multiple hypothesis testing. Controlled false discovery rate (FDR) was employed to determine the threshold of *p* values and *q* values from the corrected *p* values. Differentially expressed folds were computed as per FPKM values, that is, fold-change (FC). Screening indicators of the analysis were *p* *value* < 0.05 and log2|*FC*| > 1.

### 2.3. ceRNA Network Construction

lncRNA-miRNA pairing was performed among the screened lncRNAs in the miRcode database (http://www.mircode.org). mRNA prediction was simultaneously performed in the miRDB (http://mirdb.org/), miRTtarBase (https://miRTarBase.cuhk.edu.cn/), and TargetScan (https://www.targetscan.org/vert_80/) databases, respectively. Both lncRNA-miRNA pairing and miRNA-mRNA pairing were imported into Cytoscape (Version 3.7.2) software to construct a diagram of lncRNA-miRNA-mRNA regulatory network according to the ceRNA mechanism.

### 2.4. GO and KEGG Enrichment Analyses

The present work also tended to predict potential signaling pathways and biological functions of mRNAs and miRNAs in the ceRNA network, and GO enrichment analysis of mRNAs in ceRNAs was performed using the online tool DAVID database (https://david.ncifcrf.gov/). Also, the KOBAS database (http://kobas.cbi.pku.edu.cn/) was employed for KEGG pathway enrichment analysis, and the analysis results were summarized according to *p* < 0.05, and finally, the enrichment results were visualized in bubble chart.

### 2.5. Cell Transfection

Well-grown and stable THP-1 cells were purchased from Procell Life Science and Technology, China, and cultured using fresh RPMI1640 medium containing 100 ng/mL PMA for induction for 24 h, allowing them to differentiate into macrophages (THP-1-M0). The cells were then divided into three groups, and 1 mL of OPTI-MEM medium containing 1% si-NC plasmid and 3% Lentifusion was added to the cells in control. The culture medium of model and model + si-IL17RA-1 groups was replaced with fresh RPMI1640 medium containing 0.5 *μ*g/mL LPS and cultured for another 24 h to induce macrophage polarization to M1 type (THP-1-M1), and a sepsis model was constructed subsequently. 1 mL OPTI-MEM medium containing 1% si-NC plasmid and 3% Lentifusion was added to the cells of the model group, and 1 mL of OPTI-MEM medium containing 1% si-IL-17RA-1 plasmids and 3% Lentifusion was added into the model + si-IL-17RA-1 group. After transfection, the cells were cultured in an incubator (37°C, 5% CO_2_) for 6 days, the medium was discarded, and fresh RPMI1640 complete medium was supplemented and cultured again, followed by the harvest of cells and culture medium 48 h later. The construction of si-NC plasmids and si-IL-17RA-1 plasmids was performed by Chongqing Biomedicine Biotechnology Co. Ltd.

### 2.6. CCK-8 Assay

THP-1 cells in each group were seeded into 96 plates. Following 12 h and the supplement of 10 *μ*L CCK-8 solution (Beyotime, China) to each well, the sample cells were cultured for another 4 h. The absorbance at 450 nm was detected using an enzyme labeling reader. Cell viability (100%) = (OD experimental group − OD blank group)/(OD control group − OD blank group) × 100%.

### 2.7. Scratch Test

The THP-1 cells in each group were seeded into 6-well plates, and a scratch test was performed at a cell confluence rate of 100% using a 10 *μ*L pipette tip. Scratches were made at the center of cultural dish with even force. Following two cycles of washing with PBS, the scratched cells were removed. The wells were supplemented with 2 mL PRMI1640 complete medium. After culturing for 48 h, the results were visualized under a microscope to observe the distance of cell movement.

### 2.8. ELISA Test

The cell culture medium of each group was collected for centrifugation at 4000 rpm for 20 min, and IL-1*β*, IL-6, and TNF-*α* contents were detected as per instructions of the ELISA Kit. Human IL-1*β*, IL-6, and TNF-*α* ELISA Kits were purchased from Rui Xin Biotech Co. Ltd., China.

### 2.9. qRT-PCR

The extraction of total RNA in each group applied RNAiso Plus. Detection of the concentration and purity of RNA utilized OD1000 spectrophotometer (Thermo Fisher Scientific, USA). The reverse transcription reaction was carried out as per instructions of Goldenstar™ RT6 cDNA Synthesis Kit (Tsingke Biotechnology Co., Ltd., China). The qRT-PCR reaction was performed according to 2×T5 Fast qPCR Mix (SYBR Green I). The reaction system was 10 *μ*L of 2×T5 Fast qPCR Mix, 0.8 *μ*L of upstream and downstream primers each, 0.4 *μ*L of 50×ROX Reference Dye II, and 0.5 *μ*L of Template DNA, with a final addition of ddH_2_O to make a total volume of 20 *μ*L. A real-time quantitative PCR instrument (Thermo Fisher Scientific, USA) was applied to carry out the reaction at 95°C for 30 s and 5 s, respectively, 55°C and 72°C for 30 s, respectively, with 40 cycles in total. Primer sequences of miR-7847-3p, IL-17RA-1, PRKCG, MEKK1, and JNK are shown in Table 1.

### 2.10. Western Blot

Total protein extraction applied RIPA lysis buffer (mixed with PMSF and cocktail protease inhibitors) (Beyotime, China). Contents of proteins were detected using CBA method. After the determination of protein concentration and mixture of 500 *μ*g with 5×SDS Loading Buffer at 4 : 1, protein denaturation was performed in a heating metal bath at 100°C for 6 min. Of 20 *μ*L each, protein sample was taken for 10% SDS-PAGE electrophoresis for 90 min, delivered to a PVDF membrane, and followed by membrane blocking for 1 h at room temperature using 5% skimmed milk. By supplementation of primary antibodies, the cells were incubated at 4°C overnight. The next day, secondary antibodies were supplied and incubated for 1 h at room temperature. The membrane was evenly covered with ECL exposure solution and detected using a nucleic acid protein gel imager (Bio-Rad, USA). The gray values of bands were determined using ImageJ. Primary antibodies PRKCG (A7922), MEKK1 (A16057), JNK (A0288), p-JNK (AP0631), and GAPDH (AS014) were purchased from Abclonal, China. p-MEKK1 (PA5-40268) was from Thermo Fisher Scientific, USA.

### 2.11. Construction of Dual-Luciferase Reporter Gene Vector

Binding sites between miR-7847-3p and PRKCG gene promoter and miR-7847-3p and IL-17RA-1 were predicted on JASPAR2022 website (https://jaspar.genereg.net/). miR-7847-3p mimics was synthesized, the wild-type sequences of PRKCG (2000 bp, WT) and the mutated PRKCG sequence (2000 bp, mut) at the 3′UTR region were designed and synthesized, and wild-type IL-17RA-1 sequence (1751 bp, WT) and the mutated IL-17RA-1 sequence (1751 bp, mut) at this site were designed and synthesized. The 2000 bp sequence of PRKCG at the 3′UTR promoter region was cloned into pmiR-luc2-Rluc, a dual-luciferase reporter gene vector after synthesis, denoted as pmiR-3′UTR-WT-luc2-Rluc. Meanwhile, C1 binding site at PRKCG promoter region 2000 bp sequence was cloned into pmiR-luc2-Rluc after synthesis, denoted as pmiR-3′UTR-mut-luc2-Rluc, the IL-17RA-1 wild-type 1751 bp sequence into the pmiR-luc2-Rluc as pmiR-WT-luc2-Rluc, and C1 of 1751 bp sequence of IL-17RA-1 into pmiR-luc2-Rluc, denoted as pmiR-mut-luc2-Rluc.

### 2.12. Dual Luciferase Gene Reporter Assay

To detect the binding site between miR-7847-3p and PRKCG, the constructed plasmid vectors were mixed in the following groups, mimics NC + pmiR-NC-luc2-Rluc, mimics NC + pmiR-3′UTR-WT-luc2-Rluc, miR-7847-3p mimics + pmiR-3′UTR-WT-luc2-Rluc, miR-7847-3p mimics + pmiR-NC-luc2-Rluc, and miR-7847-3p mimics + pmiR-3′UTR-mut-luc2-Rluc. To detect the binding site between miR-7847-3p and IL-17RA-1, the constructed plasmid vectors were mixed in the following groups, mimics NC + pmiR-WT-luc2-Rluc, miR-7847-3p mimics + pmiR-WT-luc2-Rluc, miR-7847-3p mimics + pmiR-NC-luc2-Rluc, and miR-7847-3p mimics + pmiR-mut-luc2-Rluc. 1 *μ*g mixed plasmid DNA was added to 50 *μ*L of medium free from antibiotics and serum, aspirated, and mixed gently using a pipette. Following the addition of 1.6 *μ*L of nanofusion transfection reagent, the samples were mixed well, let stand at room temperature for 20 min, and cotransfect into THP-1 cells obtained by stable passage. The dual-luciferase reporter gene assay was conducted exactly as per instructions of Reporter Assay System (Promega, USA).

### 2.13. Statistical Analysis

All data were processed for analysis using one-way ANOVA and plotted using GraphPad Prism 9.0, and results were expressed as mean ± standard deviation. Differences were considered significant if *p* < 0.05 after multiple group comparisons.

## 3. Results

### 3.1. Differentially Expressed Genes (DEGs) Screening

**Figure 1:**
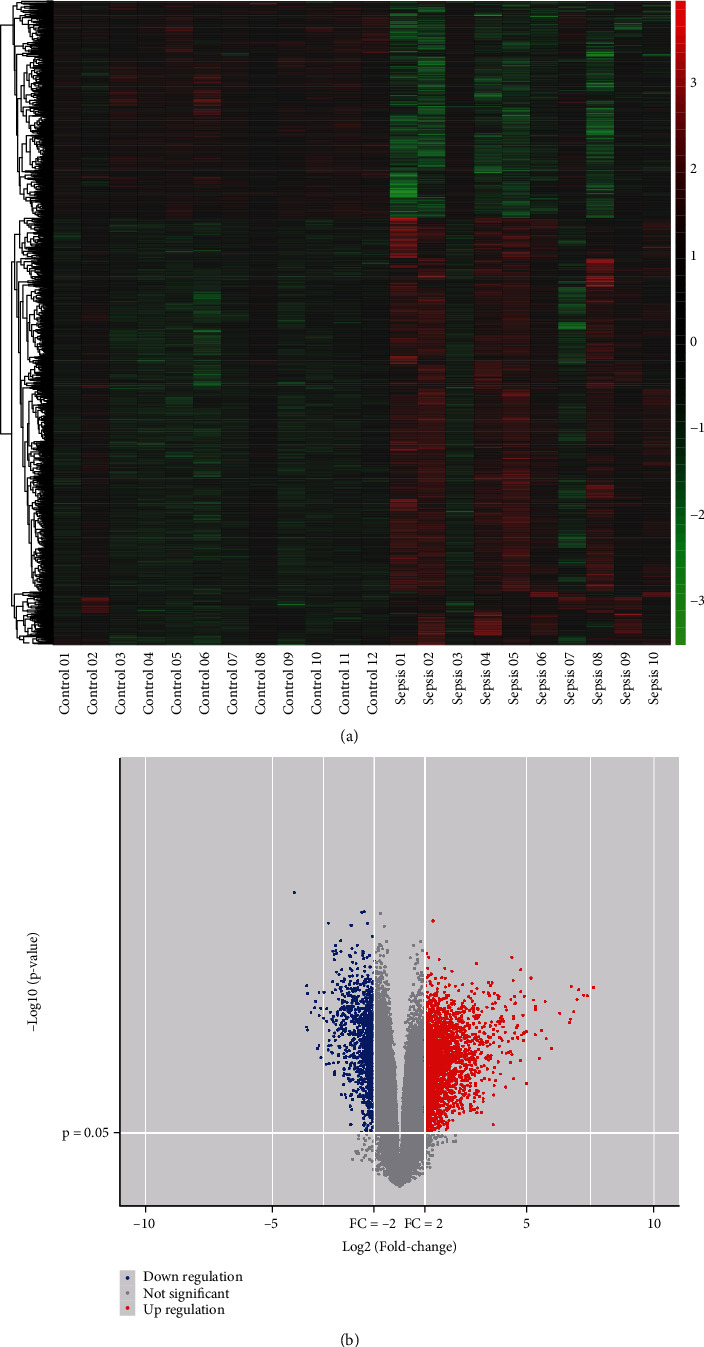
lncRNA DEGs analysis. (a) Heat map and (b) volcano map illustrations of differential genes.

**Figure 2:**
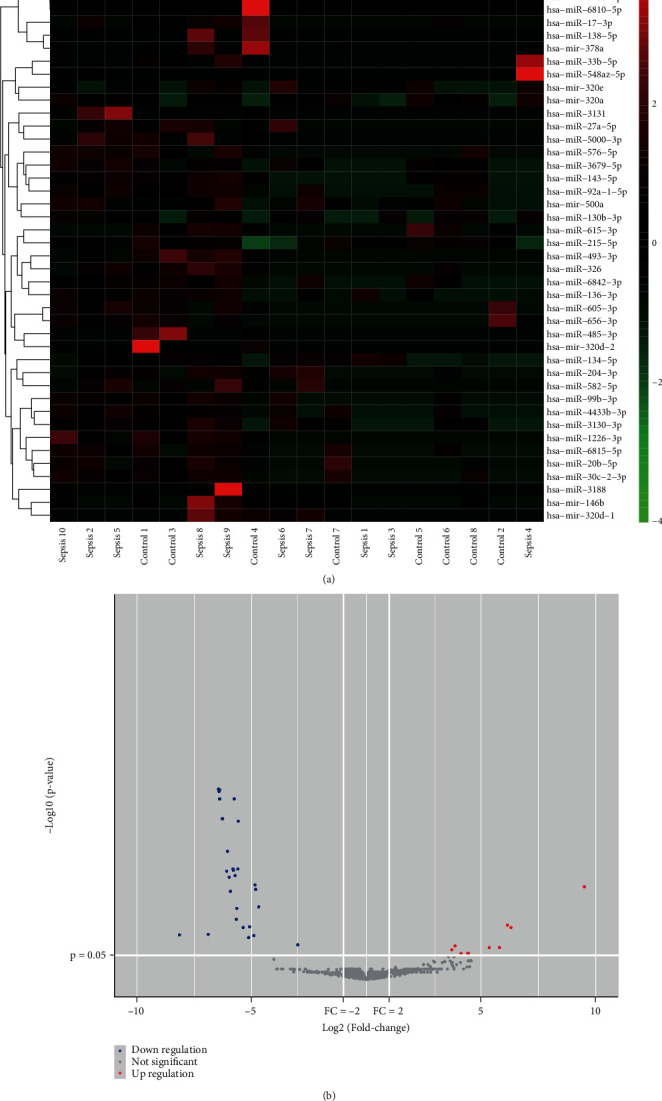
miRNA DEGs analysis. (a) Heat map and (b) volcano map illustrations of differential genes.

**Figure 3:**
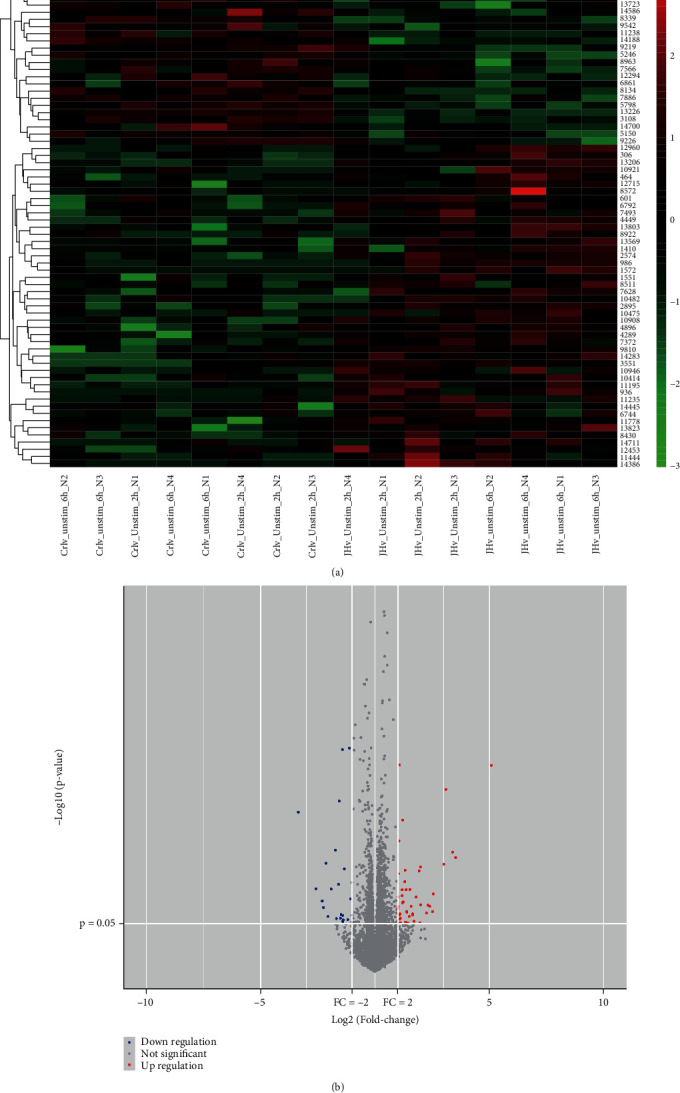
mRNA DEGs analysis. (a) Heat map and (b) volcano map illustrations of differential genes.

As per the set conditions for screening, 22 samples were collected from the GEO database, including 12 healthy controls and 10 sepsis patients. Using the screening standard *p* value < 0.05 and log2|*FC*| > 1, the number of highly expressed lncRNAs, miRNAs, and mRNAs was 2230, 11, and 45, respectively, and those poorly expressed were 1046, 32, and 24, respectively (Figures 1(a), 1(b), 2(a), 2(b), 3(a), and 3(b)).

### 3.2. ceRNA Network Construction Results

**Figure 4:**
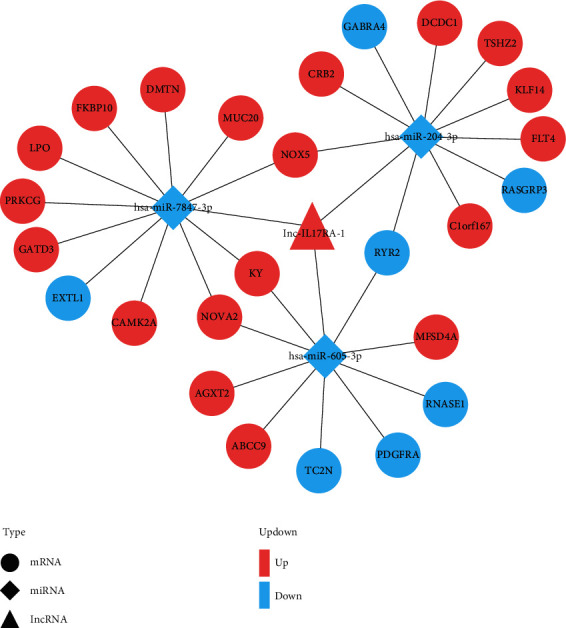
ceRNA network diagram. Triangle represents lncRNA, diamond represents miRNA, and circle represents mRNA.

The highly conserved miRcode database was employed to predict lncRNA and miRNA binding, and lncRNA-miRNA pairs were established. Subsequently, miRDB, miRTarBase, and TargetScan databases were employed for mRNA prediction to identify miRNA-mRNA relationship pairs. The previously described pairs of lncRNA-miRNA and miRNA-mRNA genes were imported into Cytoscape, and a ceRNA network diagram was constructed. The only upregulated lncRNA was IL-17RA-1. There were three downregulated miRNAs, miR-7847-3p, miR-204-3p, and miR-605-3p; nineteen upregulated mRNAs were PRKCG, LPO, FKBP10, DMTN, MUC20, NOX5, KY, NOVA2, CAMK2A, GATD3, CRB2, DCDC1, TSHZ2, KLF14, FLT4, C1orf167, MFSD4A, AGXT2, and ABCC9; and seven downregulated mRNAs were EXTL1, GABRA4, RYR2, RASGRP3, RNASE1, TC2N, and PDGFRA (Figure 4). In view of the constructed results of ceRNA network and combined with the current research topic of this experiment, we selected IL-17RA-1, PRKCG, and has-miR-7847-3p for further research.

### 3.3. GO and KEGG Enrichment Analyses

**Figure 5:**
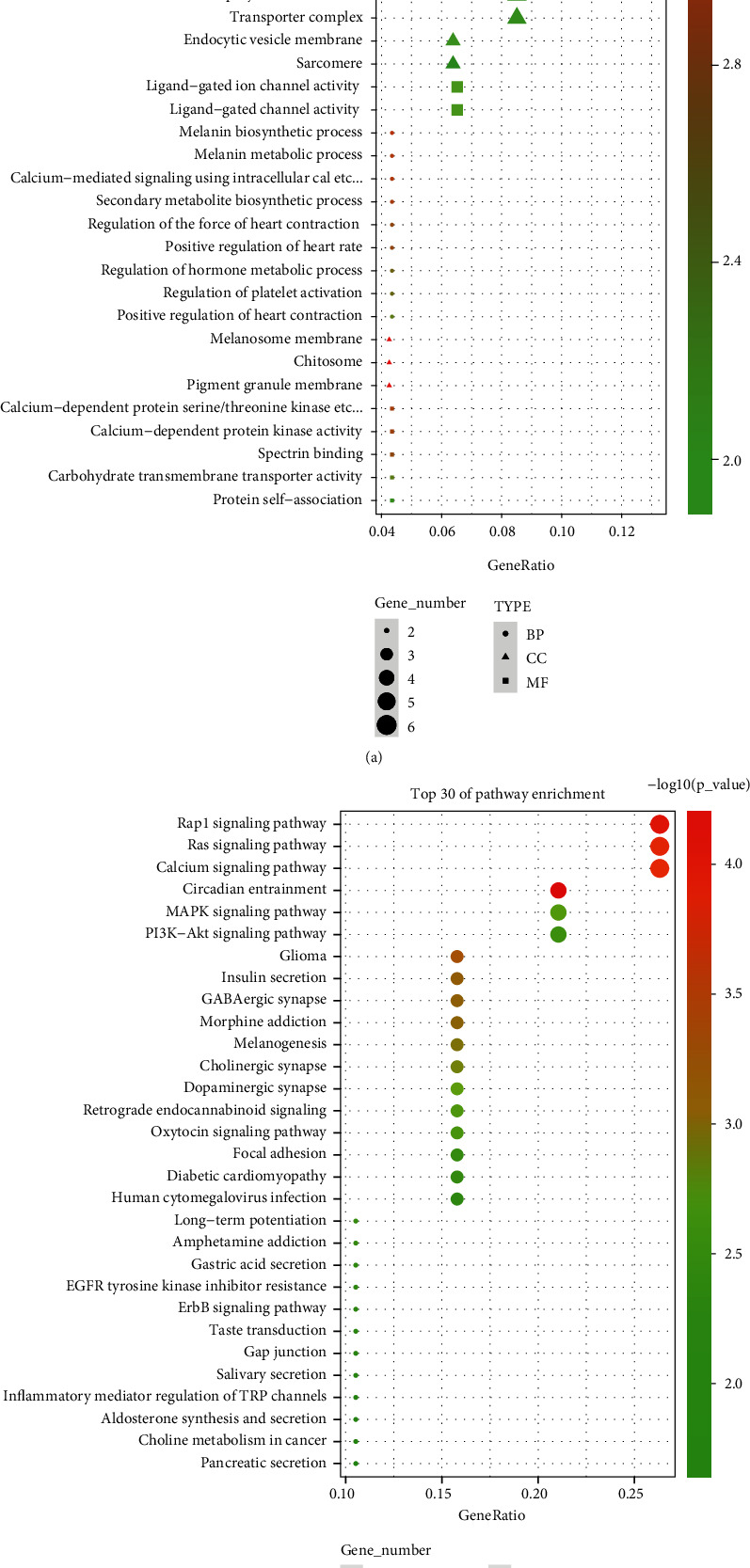
miRNA DEGs enrichment analysis. (a) GO enrichment analysis and (b) KEGG enrichment analysis.

**Figure 6:**
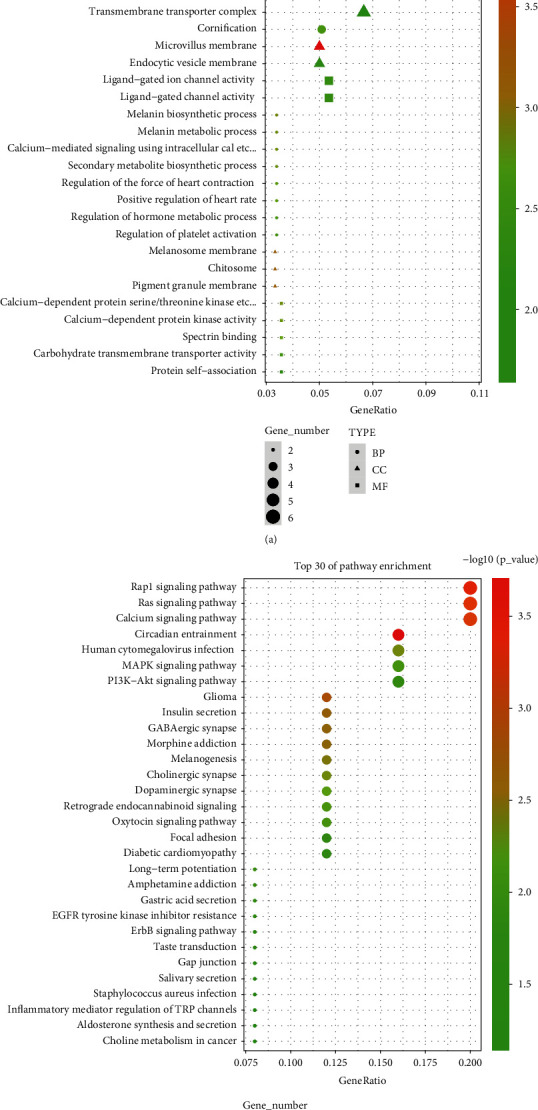
mRNA DEGs enrichment analysis. (a) GO enrichment analysis and (b) KEGG enrichment analysis.

GO function and KEGG pathway enrichment analyses were performed to clearly elucidate possible miRNA and mRNA signaling pathways associated with ceRNA network. GO functional analysis revealed that miRNAs might be involved in the biological process (BP) mainly including protein autophosphorylation. The most prominent enrichment in cellular component (CC) were ion channel complex, transmembrane transporter complex, postsynaptic specialization, cell projection membrane, and transporter complex. Enrichment results in molecular function (MF) indicated a correlation with channel activity, passive transmembrane transporter activity, and ion channel activity (Figure 5(a)). The BP that mRNA might be involved in included protein autophosphorylation and cornification, CC mainly included cell projection membrane, microvillus, and actin-based cell projection, and MF mainly included channel activity and passive transmembrane transporter activity (Figure 6(a)). The KEGG enrichment results suggested that both miRNA and mRNA might be involved in Rap1 Ras, Calcium, MAPK, and PI3K-Akt signaling pathways (Figures 5(b) and 6(b)). Based on the previously described enrichment results, combined with the research subject sepsis of this study, we selected the MAPK signaling pathway for further research.

### 3.4. The Effect of IL-17RA-1 on Cell Viability and Migration

**Figure 7:**
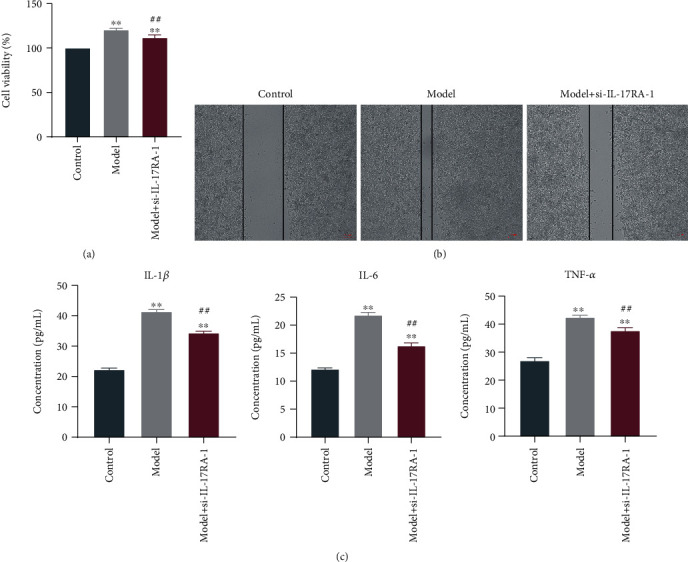
The effect of si-IL-17RA-1 on THP-1 cell viability, migration ability, and inflammatory cytokines contents in supernatant. (a) CCK-8 detection of cell viability. (b) Scratch test detection of cell migration ability. Scale bar, 100 *μ*m. (c) ELISA test detection of IL-1*β*, IL-6, and TNF-*α* contents in supernatant.

Macrophage M1 polarization is essential for sepsis occurrence and development. After PMA treatment, THP-1 cells were differentiated into macrophages, LPS was employed to induce M1-type polarization, and then the cells were transfected with si-IL-17RA-1 plasmid to test their viability and migration ability. CCK-8 analysis indicated a marked increase in cell viability of model vs. control group. After si-IL-17RA-1 transfection, the cell viability decreased greater than model (Figure 7(a)). The scratch test indicated narrower scratch width in model vs control, but that of the interference group was wider than model (Figure 7(b)). The above results indicated that IL-17RA-1 inhibited the activity of THP-1-M1, suggesting its potential role in improving sepsis.

### 3.5. The Effect of IL-17RA-1 on Proinflammatory Factors

In addition, the effect of IL-17RA-1 on the level of proinflammatory factors secreted by THP-1-M1 was further determined. The results showed that IL-1*β*, IL-6, and TNF-*α* contents of model group increased markedly vs. control and interference groups (Figure 7(c)), suggesting that IL-17RA-1 might promote the secretion of proinflammatory factors by THP-1-M1 cells, contributing to the development of sepsis.

### 3.6. The Effect of IL-17RA-1 on the MAPK Signaling Pathway

**Figure 8:**
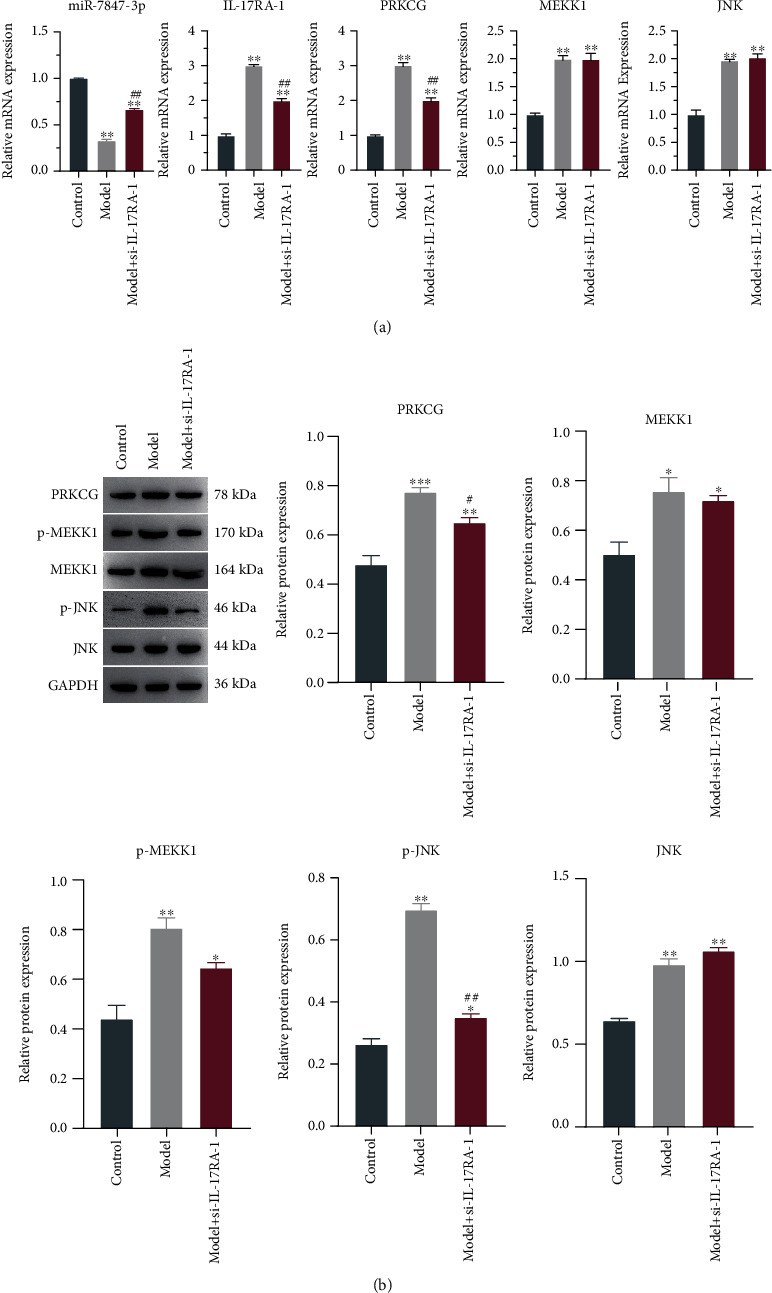
The effect of si-IL-17RA-1 on the expression of miR-7847-3p, PRKCG, MEKK1, JNK mRNA, and proteins. (a) qRT-PCR detection of mRNA expression of miR-7847-3p, IL-17RA-1, PRKCG, MEKK1, and JNK. (b) Western blot detection of PRKCG, MEKK1, JNK, p-MEKK1, and p-JNK expression.

qRT-PCR results indicated a substantial decrease in the expression of miR-7847-3p in model while a marked increase in the expression of IL-17RA-1, PRKCG, MEKK1, and JNK vs control. Conversely, miR-7847-3p was highly expressed but IL-17RA-1 and PRKCG were poorly expressed compared with model, and no significant change was found in MEKK1 and JNK (Figure 8(a)). The trends of PRKCG, MEKK1, and JNK protein expression were consistent with the qRT-PCR results, while p-JNK expression of interference group was greatly decreased as compared to model, and so did p-MEKK1 expression (Figure 8(b)). Hence, IL-17RA-1 might regulate LPS-induced sepsis cell damage by mediating miR-7847-3p and mRNA PRKCG results and activating the MAPK signaling pathway.

### 3.7. IL-17RA-1 Competitively Binds to miR-7847-3p

**Figure 9:**
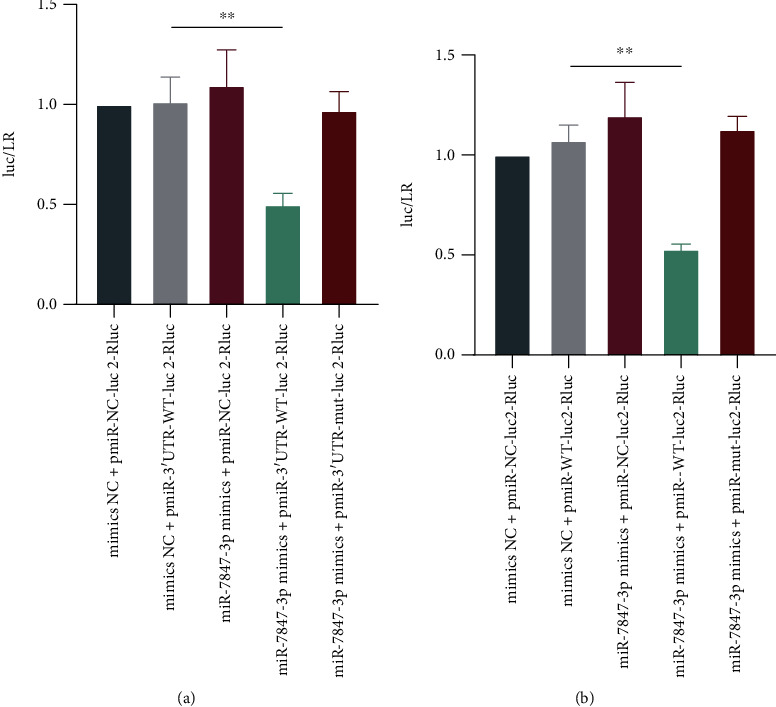
Dual-luciferase reporter gene detection of the binding sites between IL-17RA-1, miR-7847-3p, and PRKCG. (a) Binding sites detection between miR-7847-3p and PRKCG. (b) Binding sites detection between IL-17RA-1 and miR-7847-3p.

Dual-luciferase reporter gene analysis revealed that relative luciferase activity in the miR-7847-3p mimics group was greatly reduced vs. mimic NC + pmiR-3′UTR-WT-luc2-Rluc group (Figure 9(a)). However, compared with mimics NS + pmiR-WT-luc2-Rluc, the relative luciferase activity in the miR-7847-3p + pmiR-WT-luc2-Rluc group was significantly decreased (Figure 9(b)). The findings indicated that miR-7847-3p could bind to the predicted target site of 3′UTR of PRKCG gene. Moreover, IL-17RA-1 could also competitively bind to miR-7837-3p, downregulation of its expression increased the translation of PRKCG and promoted LPS-induced THP-1 cell damage.

## 4. Discussion

Sepsis is a systemic inflammatory response mediated by multiple innate immune cells, including neutrophils, monocytes, and macrophages, following severe infection [10]. Immune and inflammatory responses are vital in the pathogenesis of sepsis. Human immune cells are activated by pathogenic microorganisms and toxins to produce a variety of inflammatory cytokines, resulting in severe cell damage, microcirculatory disturbances, and organ dysfunction [11–13].

The regulation of lncRNA-miRNA-mRNA-pathways coexpression network in sepsis is a hot topic in recent years. On the one hand, the miRNA-mRNA regulatory network participates in sepsis-induced inflammatory responses. Overexpressed miR-129-5p attenuates acute lung injury induced by sepsis through targeted downregulation of HMGB1 [14]. Overexpression of miR-22-3p also suppresses acute kidney injury induced by sepsis through targeted downregulation of PTEN [15]. miR-181b and miR-21 target NFI-A in suppressor cells were derived from myeloid to enhance resistance to infection in septic mice. On the other hand, the mutual regulation between lncRNA and miRNA can affect the development of sepsis. The lncRNA MALAT1 can alleviate sepsis in burn-injured patients by regulating miR-214/TLR5 [16]; the lncRNA CRNDE is involved in aggravating the inflammatory process of sepsis through the miR-181a-5p/TLR4 axis [17]. lncRNA H19 significantly reverses sepsis-induced inflammatory response and myocardial dysfunction through miR-874/AQP1 [18].

IL-17 is differentiated from downstream Th17 cells of naive CD4+ T cells costimulated by TGF-*β* and IL-6 [19]. There are six family members of IL-17 from IL-17A to IL-17F and five IL-17 receptor family members from IL-17RA to IL-17RE [20]. Both IL-17A and IL-17F can generate homodimers or heterodimers and bind to heterodimeric IL-17RA and IL-17RC complexes, thereby activating downstream signaling transduction for host defense, autoimmune disease, or inflammation associated biological effects [21]. For example, IL-17 can act on fibroblasts, epithelial cells, and macrophages to induce various proinflammatory factors and chemokines [22]. IL-17A is also associated with inflammation by mediating the aggregation of neutrophils and macrophages [23]. It has been reported that the signal transduction intensity induced by IL-17A was positively correlated with the expression level of IL-17RA on the cell surface [24, 25]. Collectively, IL-17RA might act as a general switch to achieve various biological effects of IL-17A.

The JNK signaling pathway is an important branch of the MAPK pathway that mediates a variety of processes including cell cycle, reproduction, apoptosis, and cellular stress. Activation of JNK requires MEKK1-4 phosphorylation of MKK4/7. Previous studies showed JNK signaling pathway plays important role in sepsis. Nie et al. found that JNK selective inhibitor IQ-1S reduced the phosphorylation level of JNK2 to decrease inflammatory cytokines levels (TNF-*α*, IL-6, IL-1*β*) to protect septic mice [26]. Chang et al. demonstrated that the inhibition of p-JNK reduced the protein expression of TNF-*α* and alleviated histological myocardial injury and improved cardiac function during sepsis in mice [27]. Our experimental results are in good agreement with previous studies. After interfering with the expression of IL-17RA-1, both p-MEKK1 and p-JNK levels were significantly reduced.

Protein kinase C (PKC) is a phosphorus-dependent kinase widely distributed in mammalian eukaryotic cells. Ten PKC subclasses have been identified in mammalian tissues, including *α*, *β*1, *β*2, *γ*, *δ*, *ε*, *η*, *θ*, *ζ*, and *λ* [28]. PKC exerts its physiological effects on neurotransmitter release, synaptic shaping, cell proliferation, gene expression, cell degeneration, and programmed cell death through phosphorylation of proteins [29]. In addition, PKC, as a group of serine/threonine protein kinase family, plays an important role in many pathological processes by mediating receptor signals from extracellular sources. For example, PKC-*δ* significantly attenuates inflammatory cell infiltration, lung architecture disruption, and pulmonary edema associated with septic mice [30], and PKC-*ζ* regulates Kupffer cell apoptosis in septic rats to ameliorate liver injury [31]. Few studies have shown a role for PRC-*γ* (PRKCG) in sepsis. However, it is reported that PRKCG induced inflammation and pain in nerve injury mice [32, 33]. Based on the present results, it has been demonstrated that PRKCG also plays an important role in septic inflammation.

This paper first predicted that IL-17RA-1/miR-7847-3p/PRKCG might link to the occurrence and development of sepsis via constructing a ceRNA network diagram. Meanwhile, in vitro experiments showed that si-IL-17RA-1 inhibited the viability and migration ability of THP-1-M1 macrophages, and IL-1*β*, IL-6, and TNF-*α* levels secreted by them. Furthermore, si-IL-17RA-1 could target the upregulation of miR-7847-3p/MAPK to alleviate LPS-induced THP-1-M1 cell damage, providing a potential alternative for the prediction and prevention of sepsis.

## 5. Conclusions

This study predicted that the ceRNA network IL-17RA-1/miR-7847-3p/PRKCG might be the molecular mechanism underlying the processes of sepsis occurrence and development. And si-IL-17RA-1 was verified to inhibit LPS-induced THP-1-M1 cell viability and migration ability as well as IL-1*β*, IL-6, and TNF-*α* levels secreted by cells. IL-17RA-1 could target miR-7847-3p, which mediates PRKCG expression via MAPK signaling pathway, to alleviate sepsis response. This network might be a potential method to predict and prevent sepsis.

## Figures and Tables

**Table 1:** Primer sequences.

Gene	Sequence (5′-3′)
miR-7847-3p	F: CGTGACTGTCCCTCTGTGTC R: ATGCTGGCATTGCTCGTGG
IL-17RA-1	F: CGCTACTATGAGCGACGGTT R: GCGTGCTGCTCCATGTTATG
PRKCG	F: CAGAAGACCCGAACGGTGAA R: ATAGCGATTTCTGCCGCGTA
MEKK1	F: GATGCCAATAGCCGCACAAG R: TGTGGGGTCTGCCTTTTGTT
JNK	F: TCCTTGGCATGGGCTACAAG R: CGGGTGTTGGAGAGCTTCAT

## Data Availability

All data, models, and code generated that are used during the study appear in the submitted article.
